# Mixed endometrial carcinoma: is it time to profile the two components separately?

**DOI:** 10.3389/fonc.2026.1735988

**Published:** 2026-04-23

**Authors:** Gianna Musettini, Paola Pretelli, Andrea Giusti, Samanta Cupini, Luigi Coltelli, Filomena De Luca, Ermelinda De Maio, Chiara Finale, Luna Chiara Masini, Giulia Soria, Giovanna Cirigliano, Paolo Viacava, Sara Donati, Chiara Valsuani, C. Caparello, Maurizio Lucchesi, Ilaria Furfaro, Andrea Antonelli, Sergio Abate, Gian Luca Bracco, Roberto Marrai, M. Liut, Stefano Masoni, Camilla Pini, Marcello Mignogna, Paola Cocuzza, Alessandro Ginori, Francesca Vivaldi, Giulia Acconci, Giada Arrighi, Maria Teresa Barletta, Cecilia Barbara, Irene Stasi, Azzurra Farnesi, Andrea Marini, Antonio Pellino, E. Sammarco, Javier Rosada, Francesca Orlandi, Andrea Cavazzana, Giacomo Allegrini

**Affiliations:** 1 Division of Medical Oncology, Livorno Hospital, Department of Oncology, Azienda Local Health Unit (USL) Toscana Nord Ovest, Livorno, Italy; 2 Division of Pathological Anatomy, Carrara Hospital, Department of Diagnostic Imaging, Azienda Local Health Unit (USL) Toscana Nord Ovest, Carrara, Italy; 3 Division of Medical Oncology, Pontedera Hospital, Department of Oncology, Azienda Local Health Unit (USL) Toscana Nord Ovest, Pontedera, Italy; 4 Division of Pathological Anatomy, Livorno Hospital, Department of Diagnostic Imaging, Azienda Local Health Unit (USL) Toscana Nord Ovest, Livorno, Italy; 5 Division of Medical Oncology, Lucca Hospital, Department of Oncology, Azienda Local Health Unit (USL) Toscana Nord Ovest, Lucca, Italy; 6 Division of Medical Oncology, Pisa Hospital, Azienda Ospedaliero Universitaria Pisana, Pisa, Italy; 7 Division of Medical Oncology, Massa- Carrara Hospital, Department of Oncology, Azienda Local Health Unit (USL) Toscana Nord Ovest, Massa, Italy; 8 Division of Medical Oncology, Piombino Hospital, Department of Oncology, Azienda Local Health Unit (USL) Toscana Nord Ovest, Piombino, Italy; 9 Division of Gynecology and Obstetrics, Versilia Hospital, Maternal and Child Department, Azienda Local Health Unit (USL) Toscana Nord Ovest, Lido di Camaiore (Lucca), Italy; 10 Division of Gynecology and Obstetrics, Livorno Hospital, Maternal and Child Department, Azienda Local Health Unit (USL) Toscana Nord Ovest, Livorno, Italy; 11 Division of Gynecology and Obstetrics, Lucca Hospital, Maternal and Child Department, Azienda Local Health Unit (USL) Toscana Nord Ovest, Lucca, Italy; 12 Division of Gynecology and Obstetrics, Massa-Carrara Hospital, Maternal and Child Department, Azienda Local Health Unit (USL) Toscana Nord Ovest, Massa, Italy; 13 Division of Gynecology and Obstetrics, Pontedera Hospital, Maternal and Child Department, Azienda Local Health Unit (USL) Toscana Nord Ovest, Pontedera, Italy; 14 Division of Gynecology and Obstetrics, Cecina Hospital, Maternal and Child Department, Azienda Local Health Unit (USL) Toscana Nord Ovest, Cecina, Italy; 15 Division of Radiotherapy, Lucca Hospital, Department of Oncology, Azienda Local Health Unit (USL) Toscana Nord Ovest, Lucca, Italy; 16 Division of Pharmaceutical Prescription Appropriatness Governance, Pontedera Hospital, Department Staff Direction, Azienda Local Health Unit (USL) Toscana Nord Ovest, Pontedera, Italy; 17 Division of Internal Medicine, Livorno Hospital, Department of Internal Medicine, Azienda USL Toscana Nord Ovest, Livorno, Italy

**Keywords:** adjuvant treatment, mixed endometrial carcinoma, POLE, prognosis, serous cell carcinoma

## Abstract

**Objective:**

Advances in molecular profiling have significantly altered the approach to endometrial cancer (EC). In clinical practice, the assessment of mismatch repair (MMR) proteins and p53 status, combined with the detection of pathogenic POLE mutations, currently categorizes EC into four molecular subgroups with prognostic implications, particularly in early-stage disease: POLE-mutated, MMR-deficient (MMRd), p53-abnormal (abn), and no specific molecular profile (NSMP). However, the current approach is to assess mixed endometrial carcinomas (MEEC) as a single entity without specific molecular evaluation of individual histological components. The present study was designed as a hypothesis-generating analysis to explore this heterogeneity.

**Methods:**

The present analysis was conceived to evaluate whether profiling histological components of MEEC separately could provide additional prognostic information. MEEC with an endometrioid and a serous or clear cell component underwent immunohistochemical analysis of p53 and MMR proteins and POLE sequencing on the undissociated part and then on separate components.

**Results:**

Eight MEEC were included. Six cases of endometrioid endometrial carcinoma (EEC) and clear cell carcinoma (CCC) showed that the same mutations were detected in the undissociated tumor and in separate components. Two cases consisted of EEC with serous carcinoma (SC). Both had pathogenic POLE mutations, normal p53 expression, and pMMR status and, therefore, were potentially at low risk. Further analysis revealed differences in the histological components. In particular, in one case (case 8), the serous component was p53-abn and POLE-mutated, whereas the endometrioid component (55% of the tumor and high-grade) was POLE wild-type, representing a potential intermediate-high risk profile. It must be noted that no clinical follow-up data are available for this specific case to confirm whether this finding would have definitively altered the clinical outcome.

**Conclusion:**

Despite the retrospective nature and limited number of cases, a discrepancy was identified in a case of MEEC with a serous component when compared to molecular analysis of the tumor as a single entity, as per current guidelines. While our findings necessitate evaluation of the current molecular profiling method, the number of tumors analyzed was very restricted, and our observations pertain solely to a single sample. Therefore, these findings ought to be interpreted judiciously and are merely for the purpose of generating hypotheses.

## Highlights

- Advances in molecular profiling have transformed the management of endometrial cancer (EC), with the definition of four molecular subgroups with prognostic implications, particularly in early-stage disease: POLE-mutated, MMR-deficient (MMRd), p53-abnormal (abn), and no specific molecular profile (NSMP).
- According to the current guidelines, mixed endometrial carcinomas (MEEC) are considered as an undissociated entity, and currently no separate molecular profiling of their histological components is performed.
- This study retrospectively evaluated whether separate analysis of histological subtypes of MEEC with an endometrioid and a serous or clear cell component could provide additional prognostic information.
- In our experience, a discrepancy was identified in a case of MEEC with a serous component when compared to molecular analysis of the tumor as a single entity, and this changed the prognostic classification and consequently the therapeutic choices.

## Introduction

Endometrial carcinoma (EC) is currently the most prevalent gynecological malignancy in Europe and North America. It is the 6th most common cancer in women worldwide and the 15th most common cancer overall, with over 417,000 new cases and approximately 97,300 deaths in 2020 (1).

The most common histological type of EC is endometrioid endometrial carcinoma (EEC), accounting for 75-80% of all cases. EEC is generally characterized by a good prognosis and indolent clinical behavior.

Serous carcinoma (SC) is the second most common type of EC (approximately 10% of all cases), while clear cell carcinoma (CCC) accounts for less than 5% (approximately 2%). Both SC and CCC are clinically more aggressive, with poor chemosensitivity and a worse clinical course (2).

Mixed endometrial carcinomas (MEEC), comprising 3-10% of cases, are defined by the presence of two or more distinct histologic subtypes. Earlier definitions required the non-endometrioid component to represent at least 5% (3) of the tumor.

However, the latest WHO classification has eliminated this threshold, requiring only that the different histotypes be clearly distinguished morphologically and immunohistochemically (2). The most common MEEC types are SC combined with EEC, followed by CCC with EEC (4). In addition, other mixed histological components may also occur.

In recent years, significant advances have been made in the molecular characterization of EC. The Cancer Genome Atlas (TCGA), through the characterization of 373 ECs, identified four distinct genomic subtypes with important prognostic and potential predictive significance: “ultramutated” tumors characterized by mutations in the exonuclease domain of polymerase-ϵ (POLE), associated with an excellent prognosis (7-8%); a “copy number high” subgroup characterized by TP53 mutations and generally poor prognosis (approximately 15-20%); “hypermutated” tumors with microsatellite instability (MSI), accounting for 25-30% of cases; and “copy number-low” tumors (30-40%), with the latter two subtypes having intermediate prognosis (5).

In clinical practice, the assessment of mismatch repair (MMR) proteins and p53 status through immunohistochemistry, combined with next-generation sequencing (NGS) to identify pathogenic POLE gene mutations, currently categorizes endometrial carcinoma into four molecular subgroups: POLE-mutated, MMR-deficient (MMRd), p53-abnormal (abn), and no specific molecular profile (NSMP) (6).

The presence of a pathogenic POLE mutation seems to be associated with an excellent prognosis, regardless of classic risk factors, at least in early-stage EC.

This is also consistent with the findings of a recent meta-analysis of over 350 POLE-mutated endometrial cancers (7). Independent of traditional risk factors, such as grading, lymphovascular invasion (LVI), and myometrial involvement, these tumors have been shown to have a risk of recurrence of less than 3%, along with high and sustained salvage rates for those who experienced a disease recurrence.

According to data from the PORTEC-3 study (8), the outcome does not seem to be related to adjuvant treatment, suggesting that these patients do not benefit from receiving postoperative chemotherapy and radiation therapy.

According to the recent FIGO 2023 staging update (9) and major international guidelines (10, 11), risk stratification in EC must now include molecular classification alongside pathological risk factors.

Patients with stages I-II EC and a pathogenic POLE mutation are classified as low risk, regardless of other pathological features, and do not require any adjuvant treatment according to the ESGO/ESTRO/ESP recommendations (11). However, it should be noted that other international guidelines, such as those from the NCCN (12), maintain a more cautious approach to treatment de-escalation in this subgroup.

For stage III POLE-mutated tumors, there are currently no available outcome data without adjuvant treatment.

However, tumors with abnormal p53 and/or high-grade features, in addition to myometrial invasion (without pathogenic POLE mutation), are considered as high risk and therefore benefit from intensified adjuvant treatment with chemotherapy and radiotherapy (10–12).

The POLE mutation appears to maintain its important prognostic value even in grade 3 EECs, where they are independently associated with favorable overall survival (OS) and relapse-free survival (RFS), even in this subgroup (13, 14).

In CCC and SC (poorly differentiated histotypes by definition), molecular profiling data are limited, but POLE mutations appear uncommon (15, 16).

Thus, at least in early I and II stages, clinical decision-making is no longer based only on adverse pathological characteristics of tumors but on detailed molecular profiles. Specifically, the POLE gene plays a crucial role. This approach should be applied for all types of EC, as stated in the leading international guidelines (10–12).

MEEC are associated with a severe clinical course, with 5-year disease-free survival (DFS) and 5-year OS rates not exceeding 50% (17).

MEEC are considered as an undissociated entity whereby, following the international guidelines, current pathological diagnostics provide a histological definition of the two or more components with a unique molecular profile. In this scenario, examining the molecular components individually may provide valuable prognostic insights.

According to the current guidelines, research has not thoroughly examined the various histological categories individually and distinctly. A study published in 2020 explored eight MEEC cases (four mixed EC/SC cases, three mixed EC/CCC, and one mixed SC/CCC tumor) (18). The purpose of the analysis was to examine whether the two histological components were also distinguished by distinct molecular profiles.

In the mixed EC/CC group, additional mutations were found only in the EC component, without affecting POLE, p53, or genes of the MMR system (18). In the mixed EC/SC cases, variations were found in the expression of p53 and in the mutational status of POLE, both of which are recognized to influence prognosis and, as a result, the decision-making process.

These observations align with emerging evidence suggesting that molecular heterogeneity within mixed tumors may be more prevalent than previously assumed (18), potentially challenging the current practice of single-site profiling.

These findings could raise the question of whether the current molecular profiling, carried out by combining only the components of the two types of tissues, is no longer adequate to determine the mutational status of MEEC and, consequently, the risk of recurrence.

To assess whether molecular status stratified by histological subtypes could inform prognosis and classification, we analyzed our collection of MEEC, specifically mixed tumors with both an endometrioid component and either a CC or serous component.

This study presents the results of molecular profiling, first as an indistinct and undissociated entity (as per current clinical practice) and then by profiling the histological subtypes separately. Given the hypothesis-generating nature of this study, these findings were compared at the end of the analysis to evaluate any differences and their potential clinical significance.

## Materials and methods

We retrospectively evaluated EC cases referred to the Pathological Anatomy Unit of Azienda Usl Toscana Nord-Ovest (ATNO) from 2022 to 2024, and we selected MEEC with an endometrioid and a serous or CC component (Ethics Committee authorization CESM-AOUP, 3203/2011; EudraCT identification number: 2010-024067- 41).

Standard clinicopathologic characteristics (stage, grading, myometrial involvement, and LVI) were recorded according to the 8th edition TNM stage classification (19) and FIGO staging 2009 (20). However, risk stratification was also interpreted according to the updated FIGO 2023 criteria (9) to incorporate molecular subgroups.

We performed immunohistochemical (IHC) analysis of p53 (clone DO7, Ventana Medical Systems) and MMR proteins (MLH1, PMS2, MSH2, and MSH6) using the Ventana Benchmark Ultra-Roche Ventana platform: VENTANA anti-MLH1 (M1) Mouse Monoclonal Primary Antibody, VENTANA anti-PMS2 (A16-4) Mouse Monoclonal Antibody, VENTANA anti-MSH2 (G219-1129) Mouse Monoclonal Antibody, and VENTANA anti-MSH6 (SP93) Rabbit Monoclonal Primary Antibody. Mutation-type p53 staining was identified by either overexpression or lack of expression in tumor cell nuclei, with an intact internal control.

The p53 IHC results were interpreted as abnormal (mutated-type) staining in the presence of either strong nuclear expression in at least 80% of tumor nuclei or complete absence of expression (null-type) in 0% of tumor nuclei. This 80% threshold was selected in agreement with international consensus guidelines (22), with the purpose of improving the detection of cases exhibiting p53 abnormalities. Because commercial kits for the TP53 gene were not available during the period of this investigation, p53 immunohistochemistry was employed as a confirmed substitute for TP53 mutation status. This method has shown a general concordance of up to 92.3% in published studies on endometrial carcinoma (21).

MMR protein staining was interpreted as abnormal (loss) if any of the following criteria were met: complete loss of nuclear expression of both MLH1 and PMS2, loss of both MSH2 and MSH6, loss of MSH6 or MSH2, or loss of PMS2 or MLH1. Strong nuclear staining of normal endometrial glands, stromal cells, and lymphoid cells adjacent to the tumor was used as an internal positive control.

Following CAP and ESMO guidelines, molecular MSI analysis was adopted as a reflex test only when MMR IHC status was equivocal, defined as weak/patchy staining compared to internal controls, subclonal loss in <10% of tumor cells, absent internal controls, or predominantly non-specific cytoplasmic staining (10–23). In this study, six cases required MSI-PCR testing due to equivocal IHC results.

MSI status was assessed by the “EasyPGX^®^ ready MSI” kit PCR on “EasyPGX^®^ qPCR instrument 96” thermocycler (Diatech Pharmacogenomics) followed by analysis of denaturation profile using “Agilent Aria Software v1.4” (Agilent Technologies, Santa Clara, CA, United States) and “EasyPGX^®^ Analysis Software v4.0.0” (Diatech Pharmacogenomics, Jesi, Italy). Two mononucleotide loci (BAT25 and BAT26) and six monomorphic-nucleotide markers (NR21, NR22, NR24, NR27, CAT25, and MONO27) were analyzed; low instability (MSI-L) was defined as the presence of one unstable marker, and high instability (MSI-H) was defined as two or more markers showing an unstable melt curve profile.

DNA tumor extraction was performed from 10-µm-thick FFPE tissue sections using magnetic-particle technology “MagCore^®^ Genomic DNA FFPE One-Step Kit” on automated MagCore^®^ HF 16 Plus platform (RBC Bioscience Corp.). To ensure technical robustness, only samples with a tumor cell content >50% were selected for molecular profiling.

DNA quantity and quality were measured by qPCR on the “EasyPGX^®^ qPCR instrument 96” thermocycler; DNA input concentration and fragmentation degree were assessed using “Agilent Aria Software v1.4” (Agilent Technologies) and “EasyPGX^®^ Analysis Software v4.0.0” (Diatech Pharmacogenomics).

Mutational status of genomic DNA was assessed using the “Myriapod^®^ NGS Cancer panel DNA” kit (Diatech Pharmacogenomics) by the preparation of next generation sequencing (NGS) libraries on Illumina “MiSeq” and “iSeq 100” platforms (Illumina Inc., San Diego, CA, United States). Technical specifications for the NGS assay included a variant allele frequency (VAF) threshold of 5%, a limit of detection (LOD) >3%, and an analytical sensitivity >99%. A minimum coverage of 500X and a sequencing depth of 8,000,000 reads were maintained. Pathogenicity of the identified mutations was assessed through consultation of established databases, including ClinVar and OncoKB. Library quantification was performed on “Qubit^®^ 4.0 fluorometer” (Invitrogen by Thermo Fisher Scientific, Waltham, MA, United States).

Identification of single-nucleotide variants (SNVs) and small insertions and deletions (indels) in 17 genes (ALK, BRAF, EGFR, ERBB2, FGFR3, HRAS, IDH1, IDH2, KIT, KRAS, MET, NRAS, PDGFRA, PIK3CA, POLE, RET, and ROS1) was assessed by “Myriapod^®^ NGS Data Analysis Software v5.0.7” on “Myriapod^®^ NGS Workstation” (Diatech Pharmacogenomics).

POLE sequencing for hotspots in the exonuclease domain (exons 9–14: exon 9 D268-Q303; exon 10 G304-E340; exon 11 A341-W369; exon 13 R409-Q453; and exon 14 T454-E491) was performed by gene sequencing (panel of 17 genes) with the “Myriapod NGS Cancer Panel DNA”, CE-IVD kit (Diatech Pharmacogenetics S.r.l).

We first performed IHC and molecular analysis on undissociated MEEC, as per standard clinical practice.

Subsequently, we performed the same IHC and molecular profiling on the two components separately and distinctly (EEC/CCC or EEC/SC).

The separate and distinct extraction of DNA from the individual histological components (endometrioid and serous or CC) was performed by tissue microdissection of 10 µm thick FFPE tissue slides coated on poly-lysine glass. Unlike standard macrodissection, this microscopic architectural-guided technique was performed using a microscope (Leica DM750 Microscope integrated with 5MP CMOS camera (ICC 50W) and equipped with Leica LAS EZ software (Leica Biosystems, Wetzlar, Germany)) to ensure precise scraping of identified regions of interest (ROI) with a needle. This method allowed for high tumor DNA enrichment and accurate separation of individual histotypes, effectively avoiding cross-contamination between components.

## Results

Eight EC cases with two distinct histological components were identified: six cases comprising EEC and CCC and two cases comprising EEC and SC. The clinical, pathological, and molecular features of these tumors are summarized in **Table 1**.

**Table 1:** Clinicopathologic and molecular characteristics of MEEC and adjuvant treatment performed.

Case	Histology	Myometrial involvement (%)	LVI	Stage (TNM 8th)	Stage (FIGO ‘09)	p53	MMR	*POLE*	Adjuvant treatment
1	EEC 80%/CCC 20%	>50%	Absent	pT1bNx	IB	WT	dMMR (MSH2, MSH6)	WT	CT-RT
2	EEC 20%/CCC 80%	<50%	Absent	pT1aNx	IA	WT	pMMR	**p.S297F**	None
3	EEC 40%/CCC 60%	<50%	Absent	pT1aNx	IA	WT	dMMR (MLH1, PMS2)	WT	CT-RT
4	EEC 40%/CCC 60%	>50%	Diffuse	pT1bN0	IB	WT	pMMR	**p.V411L**	None
5	EEC 80%/CCC 20%	<50%	Absent	pT1aN0	IA	WT	dMMR (MSH6)	**p.E277K**	None
6	EEC 40%/CCC 60%	>50%	Diffuse	pT3aN0	IIIA	WT	pMMR	WT	CT-RT
7	EEC 90%/SC 10%	>50%	Diffuse	pT2N0	II	WT	pMMR	**p.A456P**	None
8	EEC 55%/SC 45%	>50%	Diffuse	pT1bN0	IB	WT	pMMR	**p.V411L**	None

EEC, endometrioid endometrial carcinoma; CCC, clear cell carcinoma; SC, serous carcinoma; LVI, lymphovascular invasion; MMR, mismatch repair; dMMR, deficient MMR; pMMR, proficient MMR; WT, wild-type; CT-RT, chemoradiation.

Values in bold represent statistically significant results (p < 0.05).

Among the six mixed EEC/CCC tumors (cases 1–6 of **Table 1**), we discovered three cases with pathogenic POLE mutations (specifically c.890C>T (p.Ser297Phe), c.890C>T (p.Ser297Phe), and c.829G>A (p.Glu277Lys), all affecting exon 9) and two tumors with dMMR, one due to loss of MSH2 and MSH6 expression and the other due to loss of MLH1 and PMS2. All cases had normal p53 expression.

**Figure 1:**
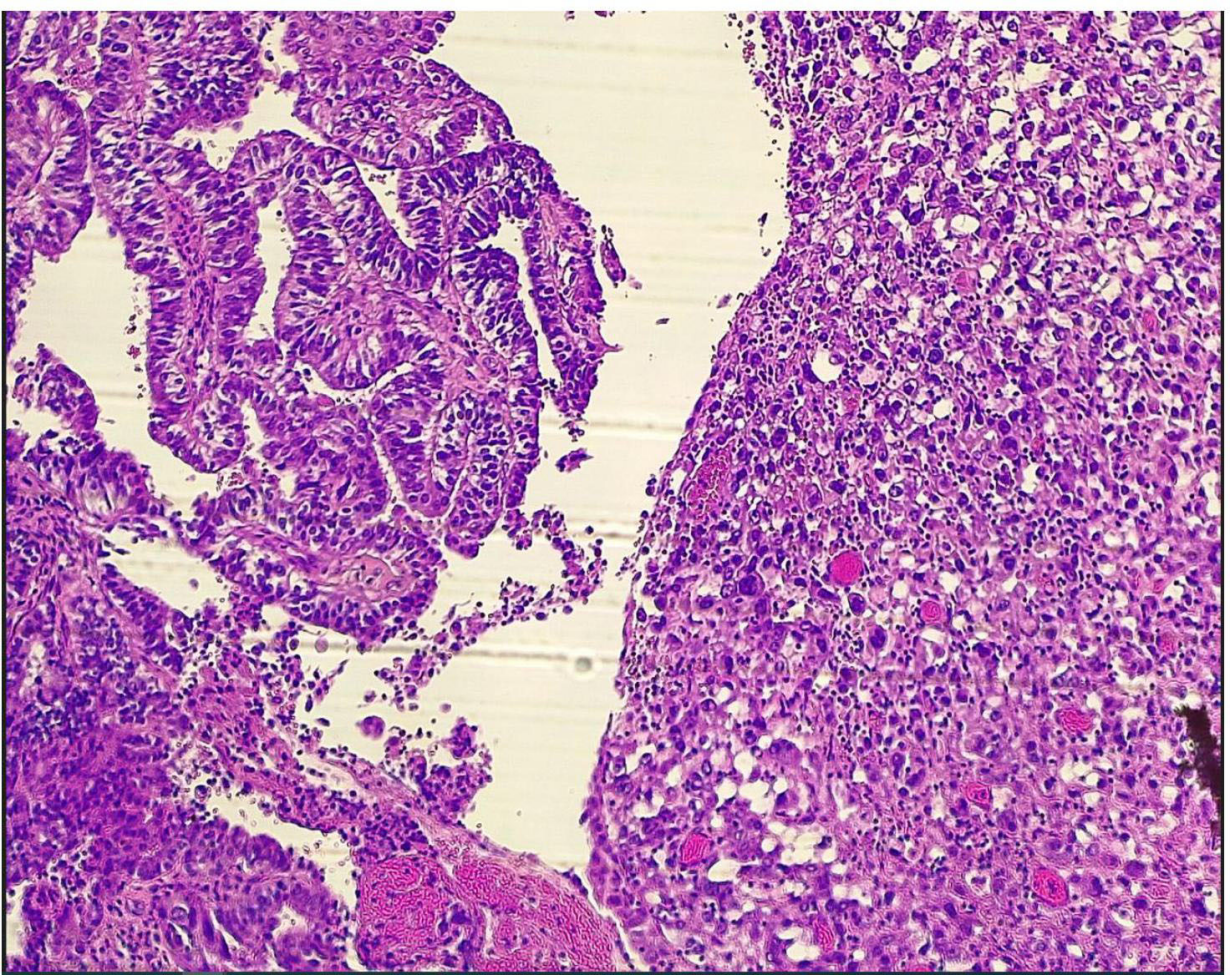
MECC with a EEC (on the right) and CCC (on the left) histological subtypes. MECC, mixed endometrial carcinoma; EEC, endometriod endometrial carcinoma; CCC, clear cell carcinoma.

The sole mixed tumor with both POLE wild-type (wt) and proficient MMR (pMMR) exhibited a BRAF mutation (c.1390G>A (p.Gly464Arg) in exon 11) and a KRAS mutation (c.38G>A (p.Gly13Asp) in exon 2). When the two components, endometrioid and clear cell, were analyzed separately (**Figure 1**), no differences were observed in their molecular profiles, which were entirely consistent with the undissociated mixed tumor (data not shown). This molecular stability across different histological areas in mixed EEC/CCC cases is consistent with findings reported by Matrai et al. (18), suggesting a shared clonal origin for these components.

**Figure 2:**
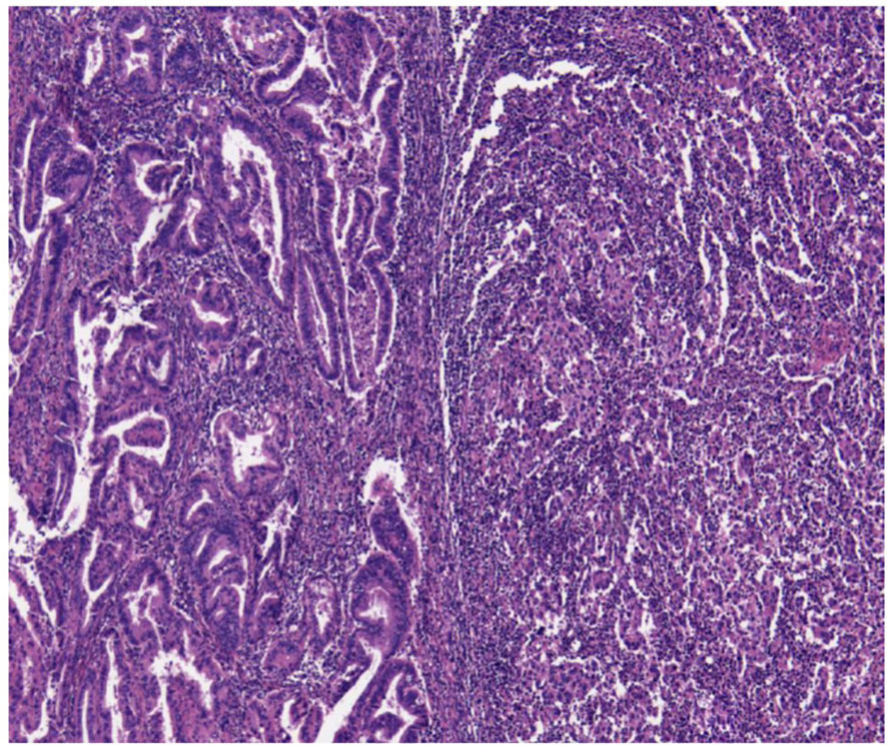
MECC with a EEC (on the right) and CCC (on the left) histological subtypes. MECC, mixed endometrial carcinoma; EEC, endometriod endometrial carcinoma; SC, serious carcinoma.

**Figure 3:**
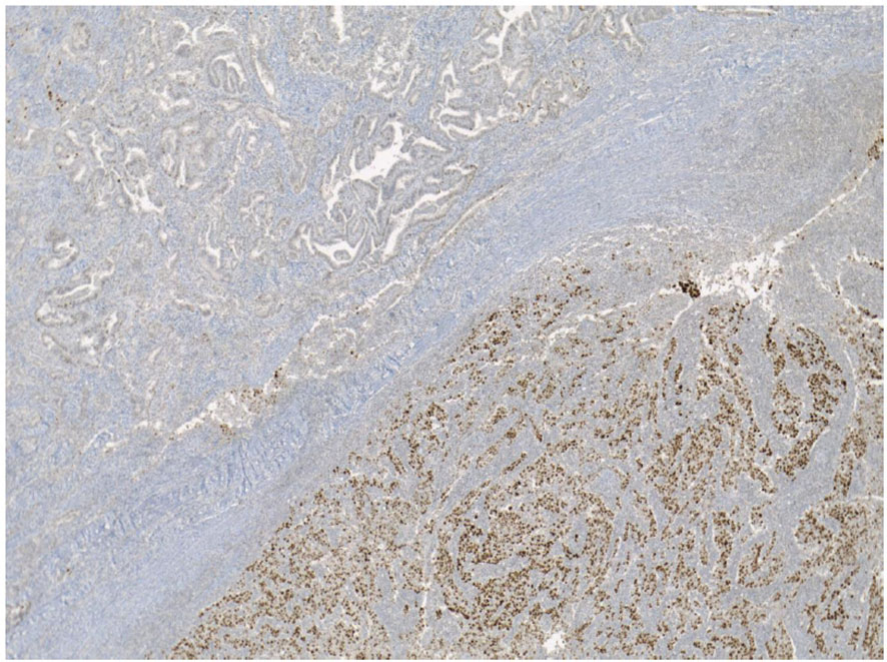
Different p53 expression in ECC (on the right) and SC (on the right) histological subtypes. EEC, endometriod endometrial carcinoma; SC, serious carcinoma.

The two combined EEC with SC (cases 7-8) were discovered to have POLE mutations (c.1366G>C (p.Ala456Pro) and c.1231G>T (p.Val411Leu) mutations, both in exon 14), normal p53 expression, and a pMMR profile. Further analysis of the molecular characteristics of the two histological components separately revealed some differences (**Figures 2**, **3**).

In case 7, the serous component was found to be p53-abn, while the endometrioid component was p53-wt. However, both subtypes presented the same pathogenic POLE mutation and a pMMR profile. The presence of a pathogenic POLE mutation affecting both histological populations confirms the excellent prognosis of the tumor, which can therefore be considered low risk according to the initial standard profiling.

Similarly, in the second tumor (case 8), the serous part was p53-abn, while the endometrioid component was p53-wt. However, while the serous subtype was confirmed to be POLE-mutated, the endometrioid component, which represents more than half of the entire tumor (approximately 55%), did not present any pathogenic mutation and was therefore POLE-wt (**Table 2**).

**Table 2:** Molecular profiling of the two mixed EEC/SC tumors: global (A) and of the two histological components separately, endometrioid (B) and serous (C).

Case	Mixed EEC/SC (A)			EEC component (B)			SC component (C)		
	**p53**	**MMR**	***POLE***	**p53**	**MMR**	***POLE***	**p53**	**MMR**	***POLE***
**7**	WT	pMMR	**p.A456P**	WT	pMMR	**p.A456P**	**ABN**	pMMR	**p.A456P**
**8**	WT	pMMR	**p.V411L**	WT	pMMR	**WT**	**ABN**	pMMR	**p.V411L**

EEC, endometrioid endometrial carcinoma; SC, serous carcinoma; MMR, mismatch repair; pMMR, proficient MMR; WT, wild-type; ABN, abnormal.

Values in bold represent statistically significant results (p < 0.05).

This is a mixed endometrial tumor in stage Ib, with diffuse LVSI. According to the standard unified profiling, it harbors a pathogenic POLE mutation and would typically be associated with a favorable prognosis, potentially leading to a recommendation for observation as the sole management strategy. However, when the two components were evaluated separately, over half of the neoplasm is represented by a POLE-wt EC, specifically a high-grade endometrioid tumor (G3). This finding in case 8 highlights a significant molecular discrepancy that contrasts with the “truncal mutation” model, in which molecular drivers are expected to be shared by all tumor areas (18).

Such discordance in POLE status within a mixed EC/SC tumor has been sporadically observed in previous literature, notably by Espinosa et al. (2020). This finding suggests an intermediate to high risk profile and might have warranted adjuvant treatment according to current clinical practice (10–12). Nevertheless, as no clinical follow-up or outcome data are available for this specific case, it cannot be definitively concluded whether this molecular heterogeneity would have translated into a different clinical course.

## Discussion

The molecular status of EC significantly changes its clinical management. Particularly in stages I and II EC, the presence of POLE pathogenic mutations confers a very good prognosis, and no further treatment is required according to ESGO/ESTRO/ESP recommendations (11), although NCCN guidelines (12) suggest a more tailored approach. Conversely, the presence of abnormal p53 or a poorly differentiated tumor subtype (without POLE mutations) suggests a more aggressive disease, and additional adjuvant therapy should be considered (5–12).

MEEC are rare and aggressive endometrial tumors. According to current guidelines, mixed tumors are considered for standard molecular profiling as a single undissociated entity. In a recent study by Wang et al., eight MEEC, including four mixed EC/SC, three mixed EC/CCC, and one mixed SC/CCC, were examined by profiling the two histological components separately and distinctly (18).

The findings showed that some tumors had components with distinct molecular profiles. Within the morphologically mixed EC/CC group, additional mutations were found only in the EC component, not affecting POLE, p53, or genes in the MMR system (18). In tumors with both endometrioid and serous components, one case exhibited a p53 mutation present only in the serous component, while another case presented a POLE pathogenic mutation only in the serous subtype. These results are consistent with our findings.

In our mixed EEC/SC cases, when profiled using a standard method as a single, undissociated entity, both cases (7 and 8) were found to be POLE mutated [c.1366G>C (p.Ala456Pro) and c.1231G>T (p.Val411Leu), respectively], p53 wild-type, and proficient mismatch repair (pMMR).

Based on the presence of a pathogenic POLE mutation and the stage (II and Ib), as stated by ESGO/ESTRO/ESP guidelines (11), both patients did not undergo additional adjuvant treatment after surgery and were referred to exclusive follow-up, despite their relatively young age and lack of other medical conditions.

In case 7, abnormal p53 expression was observed only in the serous component, but the POLE mutation persisted in both histological components, confirming the low risk of this tumor. Even based on our separate molecular profiling, the decision not to carry out any treatment remains appropriate.

In case 8, the tumor exhibited a p53 alteration only in the serous component; however, it also presented a POLE mutation, which confers a favorable prognosis.

In the EC component of case 8, which represents 55% of the total, no pathogenic POLE mutations were detected. This finding suggests a model of clonal divergence, where the POLE mutation likely emerged as a secondary, subclonal event in the serous component rather than being a “truncal” mutation shared by the entire tumor.

More than half of this mixed carcinoma consisted of an endometrioid tumor without POLE mutation but with over 50% involvement of the myometrium, high-grade, and diffuse LVSI. This type of EC might not be considered low risk but rather intermediate to high risk.

We collected these data retrospectively. If this information had been available at the time of diagnosis, it might have led to the consideration of adjuvant chemoradiotherapy treatment instead of exclusive surveillance. However, according to the standard unified profiling, this tumor had a pathogenic POLE mutation and was therefore considered to have a good prognosis and to be a candidate for exclusive follow-up (which remains the recommended approach according to standard guidelines (10–12).

It must be noted that, since no clinical follow-up or outcome data are yet available for the patients in this cohort, we cannot definitively determine whether this separate profiling would have improved the patient outcomes.

A distinct and separate molecular profiling of the two histological components of this mixed tumor, not currently indicated by any guideline or scientific community, could potentially have influenced our decision-making. Nevertheless, given the limited evidence and the exploratory nature of this case series, current data are insufficient to propose a change in clinical standards.

## Conclusions

Despite the retrospective nature and limited number of cases, a significant discrepancy was identified in a case of MEEC with a serous component when compared to the standard molecular analysis of the tumor as a single entity. Our findings suggest that separate molecular profiling, although not currently performed in clinical practice, could potentially yield different results that might more accurately reflect prognosis and influence treatment decisions. However, due to the exploratory nature of this small case series, these results should be interpreted with caution as hypothesis-generating. Further analysis involving a larger cohort is necessary to confirm whether this personalized approach provides a significant clinical benefit and justifies a change in current clinical standards.

## Limitations

This study has some limitations, including the small sample size (n=8) and its retrospective nature. Moreover, the lack of long-term follow-up and clinical outcome data, especially for case 8, prevents us from demonstrating whether separate molecular profiling would have definitively improved patient survival or changed the clinical course. Due to these constraints and the potential technical costs of separate NGS analyses, this manuscript should be considered an exploratory and hypothesis-generating case series rather than a basis for changing current clinical guidelines.

## Data Availability

The raw data supporting the conclusions of this article will be made available by the authors, without undue reservation.
